# Post-War "Nerves"

**Published:** 1921-04-02

**Authors:** 


### THE PUBLIC WELL-BEING.

### Post-War "Nerves"

"Nerves" have always been a very difficult thing to define satisfactorily, because they mean different things to different people. Before the war normal, robust, "sensible" people used to regard nervous trouble as largely one of society's make-believes, and it was gaily caricatured in the comic Press, together with its inevitable sequel the "rest-cure." There was some justification for this point of view, seeing that it appeared to be among the leisured or well-to-do classes only that the disorder manifested itself, the fact being, of course, that on the one hand a farm labourer (for instance) with "nerves" was far less likely to seek treatment or to recognise his need for it than a hard-working professional man or a woman of fashion using up a vast amount of nervous energy; while, on the other hand, the woman of fashion and the hard-working professional man were much more likely to develop nerves than the farm labourer; make-believe did no doubt play its part in pre-war "nerves," and the complaint becoming fashionable, it became customary for patients suffering from an attack of indigestion or of irritability, or both, to give the sympathetic or the cynical doctor the judicious hint as to the prescription needed.

The war, however, has changed all that. Nervous disorder is now so much of a commonplace in serious illness that people fear it as they fear any other well-defined disease, and are apt to give it even a more solemn significance than is necessary. A nervous person, in the medical sense, is recognised in the lay mind for what he is in the medical sense. A big-game hunter (said a writer the other day) who can face a lion without trepidation may not be able to travel from one station to another on the Underground. That is perfectly true, though the illustration is an extreme one, for such examples are fortunately not yet common. But what is meant is that a person may look perfectly well, and may in fact be in good physical health, and yet suffer exquisite torture through some derangement of the nervous system. The brain controls the body through the nervous system, and its functions may be seriously affected without the person affected being in the slightest degree a lunatic. In time the physical health may be seriously affected.

### Some War Experiences.

The writer, during the late war, came across many remarkable instances of what the medical service, at the instance of the military authorities, in the later period, described as "nerve failure." One case was that of an artillery gunner —a rather dull, phlegmatic fellow, of an almost gorilla-like appearance and strength, who could do amazing tricks with a heavy gun-wheel that astonished his comrades. Suddenly, for no apparent reason, he developed an uncontrollable horror of German aeroplanes. He was quite impervious to shell-fire, and had no objection to wandering about in the front-line trench system (in fact, he was subsequently only too glad to get there), but directly a German machine was signalled "over" he would behave in an extraordinary manner, falling to the ground, with his face buried in his hands, moaning and weeping, and working himself into a state of complete nervous exhaustion by the time the hum of the aeroplane had disappeared. This went on for a couple of months, his section commander being anxious to keep his condition concealed from higher authority because he had a shrewd suspicion of what the consequences might be, and hoping that the man would "get over it." On the contrary, he became, if possible, worse, and the strain so reacted upon his powerful frame that he grew as thin as a skeleton, and so weak that he was unable to lift a single round of 18-pounder ammunition. His ultimate fate was a merciful "direct hit" by enemy gunfire. But another man in the same battery—a very fine fellow, who had performed several acts of great bravery and presence of mind—developed almost precisely the same symptoms not long afterwards, and he was promptly handed over to the medical officer, and, after much difficulty, and more than one narrow escape from court-martial, he eventually reached his destination in a lunatic asylum.

### The New Treatment.

Such cases undoubtedly presented at the time a very awkward military and medical problem, but in more recent times medical science, by the aid of psychology, has been able to act with more certainty both as to diagnosis and prognosis, and wise treatment has produced what to the uninitiated must appear to be miraculous cures. In an interesting lecture a few days ago at the Institute of Hygiene Dr. J. S. Risien Russell, an authority on nervous diseases, emphasised the popular (and sometimes professional) blunder of indiscriminately labelling all such cases lunatics, and he distinguished with admirable clearness between those physical disabilities, such as paralysis, which come from organic injury to the nerve system, and those functional disorders which are equally potent, but in which there is no organic defect demonstrated, even under the microscope. As regards treatment, the victim, he points out, requires sympathy—but, we might add, not too much. He should certainly be attended to by people who know how to handle nerve cases, and who can be kind and helpful without pampering. He should be removed as far as possible from the buffets of the world and the worries of his ordinary occupation, which is the chief difficulty in a large number of cases. It is also of the greatest importance that the patient should be persuaded that he can and will recover, and this suggestion may be either directly or indirectly conveyed. Prevention being better than cure, Dr. Russell thinks that children should be carefully watched for neurotic signs, and should be taken away from school for a

**THE PUBLIC WELL-BEING**—(*continued*).

time rather than continue their education at the expense of their nervous health Very great care is required, however, not to carry this to the length of a foolish fetish. What we welcome most in this lecture is the frank way in which Dr. Russell called attention to the real danger that lurks in the rapid growth of the so-called psycho-analyist, for if it is necessary for a man to have expert knowledge to treat the body, how much more so to treat that delicate machine the mind?

The Universities and the Royal College of Physicians and Surgeons have now instituted a diploma of psychological medicine, but, beyond this, Dr. Russell considers that the Government ought to set up an inquiry into the operations of the psycho-analysts, with the object of weeding out the charlatans. That this is a matter of urgent importance must be apparent not only to members of the medical profession, but to others who are aware of the pernicious influence which the new cult is spreading among the upper and middle classes. We know, by evidence that accumulates every day from various directions, that what is euphemistically or ignorantly described as psycho-analysis threatens soon to become more generally vicious in its effect of false sexual excitations than some of the most modern manifestations of the spiritualism mania. The time to stop it is now.

